# Outpatients’ endorsement of societal recognition and respect for healthcare professionals in China’s tertiary public hospitals: a national multicenter cross-sectional study

**DOI:** 10.3389/fpubh.2026.1893761

**Published:** 2026-10-02

**Authors:** Jiarun Mi, Yuanli Liu

**Affiliations:** School of Health Policy and Management, Chinese Academy of Medical Sciences and Peking Union Medical College, Beijing, China

**Keywords:** China, healthcare professionals, outpatient experience, patient-centered care, respect, societal recognition

## Abstract

**Background:**

Societal recognition and respect for healthcare professionals are important for professional dignity and patient–clinician relationships. However, evidence remains limited on whether patients endorse healthcare professionals as deserving such recognition and respect. This study examined outpatients’ endorsement of societal recognition and respect for healthcare professionals and its associated factors in China’s tertiary public hospitals.

**Methods:**

We used data from a national multicenter cross-sectional survey conducted in the first quarter of 2021 across tertiary public hospitals in all 31 provincial-level administrative regions of mainland China. A total of 31,599 outpatients were included. The outcome was assessed using a single five-point Likert-scale normative item and dichotomized into endorsement versus non-endorsement. Mixed-effects logistic regression models with a hospital-level random intercept were used to identify associated factors. Sensitivity analyses and latent class analysis were further conducted.

**Results:**

Overall, 30,787 outpatients (97.4%) endorsed that healthcare professionals deserve societal recognition and respect. In the fully adjusted model, urban household registration, higher annual household income, and absence of chronic disease were associated with higher endorsement. Outpatient experience indicators showed the strongest associations, including satisfaction with privacy protection (OR = 3.26, 95% CI: 2.55–4.17), treatment and medication explanations (OR = 3.08, 95% CI: 2.40–3.96), registration convenience (OR = 2.38, 95% CI: 1.85–3.05), perceived respect and comfort (OR = 1.91, 95% CI: 1.47–2.49), consultation duration (OR = 1.86, 95% CI: 1.45–2.38), and waiting time (OR = 1.59, 95% CI: 1.25–2.03). Sensitivity analyses yielded consistent findings. Patients with poorer outpatient experience patterns were substantially less likely to endorse such recognition and respect.

**Conclusion:**

Most outpatients endorsed societal recognition and respect for healthcare professionals. Such endorsement was strongly linked to concrete outpatient experiences, especially privacy protection, communication, service convenience, perceived respect and comfort, consultation duration, and waiting time.

## Introduction

Societal recognition and respect for healthcare professionals constitute a relational foundation of healthcare delivery, connecting professional dignity with patient trust, quality of care, and health-system functioning (1, 2). For healthcare professionals, being valued by patients and society is closely linked to professional well-being, work engagement, and sustained commitment to care, particularly in systems facing increasing workload and workforce pressures (3–5). For patients, respectful and trusting clinical relationships are central to patient-centered care and have been associated with better healthcare experiences and health outcomes (6). Accordingly, in this study, the item is interpreted as a profession-level normative attitude regarding whether healthcare professionals, considered as an occupational group, deserve societal recognition and respect, rather than as a comprehensive multidimensional measure of societal recognition or respect (7). A useful distinction is between recognition respect and appraisal respect. Recognition respect concerns the dignity and status owed to people, whereas appraisal respect refers to esteem based on perceived qualities, conduct, achievements, or social contribution. The item used in this study is more closely aligned with recognition respect at the occupational-group level because it asks whether healthcare professionals as a group deserve societal recognition and respect, rather than asking patients to evaluate the performance or personal qualities of a particular clinician (8).

Previous studies have examined the quality of relationships between patients and healthcare professionals from multiple perspectives, with patient trust (9), satisfaction (10), patient experience (11), patient–clinician communication (12), and patient-centered care (7) being among the most frequently investigated themes. Patients’ evaluations of healthcare services are shaped not only by clinical outcomes, but also by the quality of communication, perceived respect, adequacy of information provision, emotional support, and the broader experience of care delivery (6, 11). Trust and respect toward healthcare professionals are also key components of constructive patient–clinician relationships and have been linked to treatment adherence, patient satisfaction, care experience, and evaluations of healthcare quality (1). However, existing research has largely focused on whether patients trust clinicians, are satisfied with healthcare services, or feel respected during care; comparatively less empirical attention has been paid to whether patients further endorse healthcare professionals, as an occupational group, as deserving societal recognition and respect, particularly in large national multicenter outpatient populations.

In this study, societal recognition and respect refer to a profession-level normative judgment about whether healthcare professionals, as an occupational group, deserve social appreciation and dignity. These concepts should be distinguished from related constructs. Societal appreciation of the profession refers to recognition of the social contribution and value of healthcare work, whereas interpersonal respect refers to whether a patient is treated with dignity during a specific clinical encounter. Professional prestige refers to the status or esteem attached to an occupation; trust refers to confidence in a particular healthcare professional’s competence, intentions, or reliability; reciprocity refers to mutual recognition and obligations between patients and healthcare professionals; and patient satisfaction refers to an evaluation of the care or service received. Patient-centered care refers to a broader approach to care that responds to patients’ needs, preferences, and values, whereas feeling respected by an individual clinician refers to a patient’s experience of being treated with dignity during a specific interaction. Although these domains may be related to patients’ endorsement of societal recognition and respect, they are conceptually distinct and were not separately measured by the single item used in this study.

Outpatients represent one of the most direct and frequent points of contact between the public and the healthcare system, and their views of the professional value of healthcare professionals are often grounded in concrete care-seeking experiences (13). Compared with inpatient care, outpatient services are typically characterized by high patient volume, condensed care processes, relatively brief patient-clinician encounters, and rapidly formed service impressions (14). Patients often move through a sequence of service steps within a limited period, from preparation for consultation and on-site waiting to clinical communication and subsequent care arrangements (15). Outpatient experience is not determined solely by interpersonal interactions with clinicians. It is also shaped by how effectively the health system allocates and transforms available resources into accessible, timely, efficient, and appropriate services. Accordingly, registration convenience, waiting time, consultation duration, treatment and medication explanations, privacy protection, and perceived respect may reflect both patient-clinician interactions and broader organizational and system-level processes (16). These organizational conditions may be influenced by financial constraints, institutional priorities, workforce pressures, and broader health-system disruptions, which can affect staffing, appointment and registration systems, waiting times, consultation time, privacy arrangements, and the continuity and appropriateness of services. Consequently, patient experience may reflect the organizational and financial conditions under which care is delivered, rather than individual clinician behavior alone (17). In this context, outpatients’ endorsement that healthcare professionals deserve societal recognition and respect may offer insight into how immediate, time-constrained clinical encounters shape patients’ broader social evaluation of healthcare professionals.

The study addresses this gap in three ways. First, it shifts the focus from evaluations of a particular clinician or care encounter, including trust, satisfaction, and feeling respected, to patients’ broader social evaluation of healthcare professionals as an occupational group. Second, it examines factors associated with this profession-level normative endorsement across individual sociodemographic, health, and economic characteristics, hospital-level factors, and outpatient process and humanistic care experiences. Third, it complements variable-centered regression analyses with latent class analysis to identify complex profiles of outpatient experience and examine their associations with endorsement. Sensitivity analyses were also conducted to assess the robustness of the findings.

## Methods

### Study design and population

Our study used a national, multicenter, cross-sectional survey design to examine outpatients’ endorsement that healthcare professionals deserve societal recognition and respect and its associated factors in China. Data were obtained from the outpatient survey of the fifth National Third-Party Evaluation of the Action Plan for Further Improving Healthcare Services, which was commissioned by the National Health Commission of China and the National Administration of Traditional Chinese Medicine and implemented by the School of Health Policy and Management, Peking Union Medical College (18). The survey was conducted in the first quarter of 2021 and covered tertiary public hospitals across all 31 provincial-level administrative regions of mainland China.

Briefly, a two-stage sampling design was adopted. In the first stage, hospitals were selected to ensure coverage of all 31 provincial-level administrative regions of mainland China and major types of tertiary public hospitals. In each provincial-level administrative region, one general hospital, one traditional Chinese medicine hospital, and one maternal and child health hospital were randomly selected, yielding 93 local tertiary public hospitals. Additionally, 43 tertiary hospitals affiliated with or administered by the former national health administrative authority and seven traditional Chinese medicine hospitals directly administered by the National Administration of Traditional Chinese Medicine were included. This resulted in a final sample of 143 tertiary public hospitals, including general hospitals, traditional Chinese medicine hospitals, maternal and child health hospitals, and other specialty hospitals.

In the second stage, trained third-party investigators recruited eligible outpatients on site at designated outpatient service areas. According to the evaluation protocol, the minimum required sample size for each hospital was calculated based on an expected outpatient satisfaction rate of 85%, a significance level of 0.05, and an allowable error of 0.05, yielding a minimum sample size of 196 outpatients per hospital. To allow for non-response and data cleaning, each sampled hospital was required to complete at least 200 valid outpatient questionnaires. Eligible participants were outpatients who had completed all or most of their outpatient care process on the survey day; patients attending emergency departments, special outpatient clinics, or international medical departments were not included. For patients who were unable to complete the questionnaire independently, such as older adults or children, guardians or accompanying family members were allowed to respond on their behalf.

The outpatient questionnaire was developed by the School of Health Policy and Management, Peking Union Medical College, through iterative revision based on literature review, expert consultation, stakeholder discussions, and pilot testing. The survey instrument was reviewed by experts to ensure content validity and has demonstrated acceptable validity and reliability. Psychometric assessment showed that all item factor loadings exceeded 0.50 in exploratory factor analysis, indicating acceptable construct validity, and Cronbach’s alpha coefficients for each dimension exceeded 0.75, indicating good internal consistency (19). Before the survey, provincial expert teams and investigators received standardized training on the survey protocol, item definitions, and questionnaire administration. During data collection, provincial expert teams were responsible for on-site guidance, progress monitoring, and quality control. Investigators were instructed to administer the questionnaire in a standardized and neutral manner, ensure the completeness of responses, and minimize information bias during data collection.

A total of 39,870 outpatients were approached, including 5,849 who declined participation. The remaining 34,021 outpatients completed and returned the questionnaire, yielding a response rate of 85.3%. Among the returned questionnaires, 31,622 met the prespecified validity criteria, corresponding to a questionnaire validity rate of 92.9%. For the current analysis, 23 respondents with missing information on the outcome variable were further excluded, resulting in a final analytical sample of 31,599 outpatients. This study was approved by the Ethics Committee (Approval No. CAMS&PUMC-IEC-2021-033).

### Study variables

The outcome variable was outpatients’ endorsement that healthcare professionals deserve societal recognition and respect. It was derived from the outpatient questionnaire item: “I think healthcare professionals deserve societal recognition and respect.” Conceptually, this item was treated as a single-item, profession-level normative indicator. It asks whether healthcare professionals as an occupational group deserve societal recognition and respect. It should not be interpreted as a measure of trust in a particular healthcare professional, patient satisfaction, patient-centered care, or whether the patient feels respected by an individual clinician during a specific clinical encounter. It also does not separately measure interpersonal respect, professional prestige, or reciprocity. Accordingly, endorsement was interpreted as agreement with this specific statement rather than as a multidimensional score of societal recognition and respect. Participants responded on a five-point Likert scale: “strongly disagree,” “disagree,” “neutral,” “agree,” and “strongly agree.” All five categories were retained for descriptive analyses. For the primary binary outcome, “agree” and “strongly agree” were classified as endorsement because they represent explicit agreement with the statement. “Neutral,” “disagree,” and “strongly disagree” were classified as non-endorsement because they do not represent explicit endorsement. Thus, non-endorsement in the primary analysis indicates the absence of explicit agreement and does not imply that all respondents selecting “neutral” actively rejected the statement. This definition was chosen to align the primary outcome with the study objective of identifying factors associated with patients’ explicit endorsement that healthcare professionals deserve societal recognition and respect.

Covariates were selected from the outpatient questionnaire and hospital survey information. Patient sociodemographic characteristics included age group, sex, senior high school education or above, household registration status, and annual household income. Health status and economic factors included presence of chronic disease, total cost of this outpatient visit, and perceived out-of-pocket financial burden. Outpatient process and humanistic care experience variables were derived from patients’ evaluations of their care-seeking process and interpersonal experience during the visit, including satisfaction with registration convenience, waiting time, consultation duration, treatment and medication explanations, perceived respect and comfort from healthcare professionals, and satisfaction with privacy protection.

Hospital-level variables were obtained from the institutional information collected in the national evaluation, including hospital type and geographic region of hospital. Hospital type was categorized as general hospital, traditional Chinese medicine hospital, maternal and child health hospital, or other specialty hospital. Geographic region was classified as eastern, central, or western China according to the location of the sampled hospital.

### Statistical analysis

Descriptive analyses were first conducted to summarize participant characteristics according to the original five response categories of outpatients’ endorsement that healthcare professionals deserve societal recognition and respect: “strongly disagree,” “disagree,” “neutral,” “agree,” and “strongly agree.” Categorical variables were presented as numbers and percentages.

For regression analyses, the outcome was dichotomized according to the study objective. The primary mixed-effects logistic regression model used the binary outcome defined above. Because 97.4% of respondents were classified as endorsing the statement and only 2.6% as non-endorsing, the outcome distribution was highly imbalanced. Univariable mixed-effects logistic regression models were first fitted to estimate crude associations between each factor and endorsement. Because outpatients were nested within hospitals, a hospital-level random intercept was included to account for within-hospital clustering (20). Associations were reported as odds ratios (ORs) with 95% confidence intervals (CIs).

Sequential multivariable mixed-effects logistic regression models were then constructed to examine the associations between individual-level factors and endorsement. Model 1 included patient sociodemographic characteristics, including age, sex, senior high school education or above, household registration status, and annual household income. Model 2 additionally adjusted for hospital characteristics, including hospital type and geographic region of hospital. Model 3 further included health status and economic factors, including presence of chronic disease, total cost of this outpatient visit, and perceived out-of-pocket financial burden. Model 4 additionally included outpatient process and humanistic care experience, including satisfaction with registration convenience, waiting time, consultation duration, treatment and medication explanations, perceived respect and comfort from healthcare professionals, and satisfaction with privacy protection. All multivariable models included a hospital-level random intercept. Multicollinearity among the six outpatient process and humanistic-care indicators was assessed using variance inflation factors (VIFs) derived from the fully adjusted mixed-effects logistic regression model. VIF values below 5 were considered indicative of no concerning multicollinearity. To quantify hospital-level clustering, we extracted the variance of the hospital-level random intercept from the fully adjusted mixed-effects logistic regression model. The intraclass correlation coefficient (ICC) was calculated as ICC = σ²hospital/(σ²hospital + π²/3).

Four sensitivity analyses were conducted to evaluate the robustness of the main findings from complementary perspectives. First, because the main binary outcome classified “neutral” responses as non-endorsement, we excluded participants who selected “neutral” and refitted the mixed-effects logistic regression models. This analysis was intended to assess whether the observed associations were sensitive to the potentially ambiguous interpretation of neutral responses. Second, we fitted conventional logistic regression models without hospital-level random intercepts to examine whether the results depended on the multilevel modeling strategy used to account for within-hospital clustering. Consistency between these models and the main mixed-effects models would suggest that the findings were not primarily driven by the specification of hospital-level random effects. Third, to avoid potential information loss from dichotomizing the original five-level Likert response, we fitted mixed-effects ordinal logistic regression models with a hospital-level random intercept using the original ordered outcome categories of “strongly disagree,” “disagree,” “neutral,” “agree,” and “strongly agree.” This analysis assessed whether the main conclusions were robust when the ordinal nature of the outcome was preserved. Fourth, we excluded questionnaires completed by proxy respondents and refitted the fully adjusted mixed-effects logistic regression model among questionnaires completed by patients themselves.

To complement the variable-centered regression analyses, we further conducted latent class analysis (21) as a person-centered approach to characterize overall outpatient experience patterns. The analysis was based on six outpatient process and humanistic care indicators: satisfaction with registration convenience, waiting time, consultation duration, treatment and medication explanations, perceived respect and comfort from healthcare professionals, and satisfaction with privacy protection. Each indicator was recoded as a binary variable before the LCA. Responses classified as “No” were coded as 1 and corresponded to original responses from 1 to 3, whereas responses classified as “Yes” were coded as 2 and corresponded to original responses from 4 to 5. Latent class models with one to five classes were estimated using the poLCA package. The one-class model was fitted using one initialization, whereas each model with two to five classes was fitted using 30 random initializations. A fixed random seed of 2026 and a maximum of 5,000 iterations were used for each model. Models with one to five latent classes were compared using the Akaike information criterion, Bayesian information criterion, entropy, latent class proportions, and substantive interpretability. The four-class model was selected based on the lowest Bayesian information criterion, good entropy, acceptable class proportions, and clear interpretability. After assigning participants to their most likely latent class, mixed-effects logistic regression models with a hospital-level random intercept were fitted to examine the association between latent outpatient experience patterns and endorsement.

All analyses were conducted using R software (version 4.2.0). All statistical tests were two-sided, and *p* values <0.05 were considered statistically significant.

## Results

### Participant characteristics

A total of 31,599 outpatients were included. Among the 31,599 questionnaires included in the analysis, 25,726 (81.4%) were completed by patients themselves, whereas 5,873 (18.6%) were completed by proxy respondents. Overall, 24,077 (76.2%) strongly agreed and 6,710 (21.2%) agreed that healthcare professionals deserve societal recognition and respect, while 703 (2.2%) were neutral, 79 (0.3%) disagreed, and 30 (0.1%) strongly disagreed. Thus, 30,787 participants (97.4%) expressed endorsement. The distribution was therefore highly concentrated in the endorsement category, with only 812 participants (2.6%) classified as non-endorsement. This pattern indicates a pronounced ceiling effect and substantial imbalance in the outcome. Most participants were aged 18–35 years (44.1%) or 36–59 years (34.1%); 66.4% were female, 79.2% had senior high school education or above, and 71.2% had urban household registration. General hospitals accounted for the largest proportion of participants (40.6%), and nearly half were from hospitals in eastern China (47.7%). In terms of outpatient experience, most participants reported positive evaluations, including satisfaction with registration convenience (95.0%), waiting time (90.3%), consultation duration (91.9%), treatment and medication explanations (94.7%), perceived respect and comfort from healthcare professionals (95.1%), and privacy protection (95.4%) (Table 1).

**Table 1:** Characteristics of outpatients according to their endorsement of societal recognition and respect for healthcare professionals.

Characteristic	Strongly disagree (*N* = 30, 0.1%)	Disagree (*N* = 79, 0.3%)	Neutral (*N* = 703, 2.2%)	Agree (*N* = 6,710, 21.2%)	Strongly agree (*N* = 24,077, 76.2%)	Overall (*N* = 31,599)
Age						
≤17 years	4 (0.2%)	7 (0.3%)	59 (2.3%)	554 (21.7%)	1,925 (75.5%)	2,549 (8.1%)
18–35 years	12 (0.1%)	29 (0.2%)	310 (2.2%)	2,901 (20.9%)	10,650 (76.6%)	13,902 (44.1%)
36–59 years	9 (0.1%)	28 (0.3%)	239 (2.2%)	2,313 (21.5%)	8,145 (75.9%)	10,734 (34.1%)
≥60 years	4 (0.1%)	14 (0.3%)	93 (2.1%)	919 (21.2%)	3,297 (76.2%)	4,327 (13.7%)
Missing	1 (1.1%)	1 (1.1%)	2 (2.3%)	23 (26.4%)	60 (69.0%)	87 (0.3%)
Sex						
Female	20 (0.1%)	45 (0.2%)	447 (2.1%)	4,346 (20.7%)	16,123 (76.8%)	20,981 (66.4%)
Male	10 (0.1%)	34 (0.3%)	256 (2.4%)	2,364 (22.3%)	7,954 (74.9%)	10,618 (33.6%)
Senior high school education or above						
No	10 (0.2%)	19 (0.3%)	154 (2.3%)	1,598 (24.3%)	4,807 (73.0%)	6,588 (20.8%)
Yes	20 (0.1%)	60 (0.2%)	549 (2.2%)	5,112 (20.4%)	19,270 (77.0%)	25,011 (79.2%)
Household registration status						
Rural	11 (0.1%)	30 (0.3%)	259 (2.8%)	2,151 (23.6%)	6,646 (73.1%)	9,097 (28.8%)
Urban	19 (0.1%)	49 (0.2%)	444 (2.0%)	4,559 (20.3%)	17,431 (77.5%)	22,502 (71.2%)
Annual household income						
≤60,000 yuan	16 (0.1%)	53 (0.4%)	406 (3.0%)	3,243 (24.1%)	9,730 (72.4%)	13,448 (42.6%)
60,000–120,000 yuan	2 (0.0%)	11 (0.1%)	150 (2.0%)	1,612 (21.1%)	5,881 (76.8%)	7,656 (24.2%)
≥120,000 yuan	12 (0.1%)	15 (0.1%)	147 (1.4%)	1,855 (17.7%)	8,466 (80.7%)	10,495 (33.2%)
Hospital type						
General hospital	9 (0.1%)	36 (0.3%)	267 (2.1%)	2,460 (19.2%)	10,068 (78.4%)	12,840 (40.6%)
Traditional Chinese medicine hospital	9 (0.1%)	22 (0.3%)	190 (2.3%)	2,094 (25.8%)	5,809 (71.5%)	8,124 (25.7%)
Maternal and child health hospital	11 (0.2%)	12 (0.2%)	187 (2.6%)	1,596 (21.8%)	5,507 (75.3%)	7,313 (23.1%)
Other specialty hospital	1 (0.0%)	9 (0.3%)	59 (1.8%)	560 (16.9%)	2,693 (81.1%)	3,322 (10.5%)
Geographic region of hospital						
Eastern	5 (0.0%)	34 (0.2%)	293 (1.9%)	2,908 (19.3%)	11,827 (78.5%)	15,067 (47.7%)
Central	11 (0.2%)	12 (0.2%)	147 (2.1%)	1,398 (19.8%)	5,505 (77.8%)	7,073 (22.4%)
Western	14 (0.1%)	33 (0.3%)	263 (2.8%)	2,404 (25.4%)	6,745 (71.3%)	9,459 (29.9%)
Presence of chronic disease						
No	23 (0.1%)	43 (0.2%)	473 (2.0%)	4,842 (20.9%)	17,818 (76.8%)	23,199 (73.4%)
Yes	7 (0.1%)	36 (0.4%)	230 (2.7%)	1,868 (22.2%)	6,259 (74.5%)	8,400 (26.6%)
Perceived out-of-pocket financial burden						
No	17 (0.1%)	46 (0.2%)	437 (2.2%)	3,803 (19.5%)	15,221 (78.0%)	19,524 (61.8%)
Yes	13 (0.1%)	33 (0.3%)	266 (2.2%)	2,907 (24.1%)	8,856 (73.3%)	12,075 (38.2%)
Satisfied with registration convenience						
No	10 (0.6%)	32 (2.0%)	226 (14.3%)	608 (38.5%)	702 (44.5%)	1,578 (5.0%)
Yes	20 (0.1%)	47 (0.2%)	477 (1.6%)	6,102 (20.3%)	23,375 (77.9%)	30,021 (95.0%)
Satisfied with waiting time						
No	11 (0.4%)	32 (1.0%)	297 (9.7%)	1,089 (35.5%)	1,637 (53.4%)	3,066 (9.7%)
Yes	19 (0.1%)	47 (0.2%)	406 (1.4%)	5,621 (19.7%)	22,440 (78.6%)	28,533 (90.3%)
Satisfied with consultation duration						
No	14 (0.5%)	38 (1.5%)	294 (11.5%)	950 (37.2%)	1,261 (49.3%)	2,557 (8.1%)
Yes	16 (0.1%)	41 (0.1%)	409 (1.4%)	5,760 (19.8%)	22,816 (78.6%)	29,042 (91.9%)
Satisfied with treatment and medication explanations						
No	13 (0.8%)	42 (2.5%)	276 (16.6%)	668 (40.2%)	662 (39.9%)	1,661 (5.3%)
Yes	17 (0.1%)	37 (0.1%)	427 (1.4%)	6,042 (20.2%)	23,415 (78.2%)	29,938 (94.7%)
Perceived respect and comfort from healthcare professionals						
No	10 (0.6%)	37 (2.4%)	265 (17.0%)	664 (42.5%)	586 (37.5%)	1,562 (4.9%)
Yes	20 (0.1%)	42 (0.1%)	438 (1.5%)	6,046 (20.1%)	23,491 (78.2%)	30,037 (95.1%)
Satisfied with privacy protection						
No	6 (0.4%)	37 (2.6%)	258 (18.0%)	557 (38.9%)	574 (40.1%)	1,432 (4.6%)
Yes	17 (0.1%)	41 (0.1%)	430 (1.5%)	5,926 (20.1%)	23,004 (78.2%)	29,418 (95.4%)
Missing	7 (0.9%)	1 (0.1%)	15 (2.0%)	227 (30.3%)	499 (66.6%)	749 (2.4%)

### Univariate analyses

As shown in Figure 1, univariable mixed-effects logistic regression models were fitted to estimate crude ORs for each factor. These models included a hospital-level random intercept but were not adjusted for other covariates; therefore, the estimates represent crude associations.

**Figure 1:**
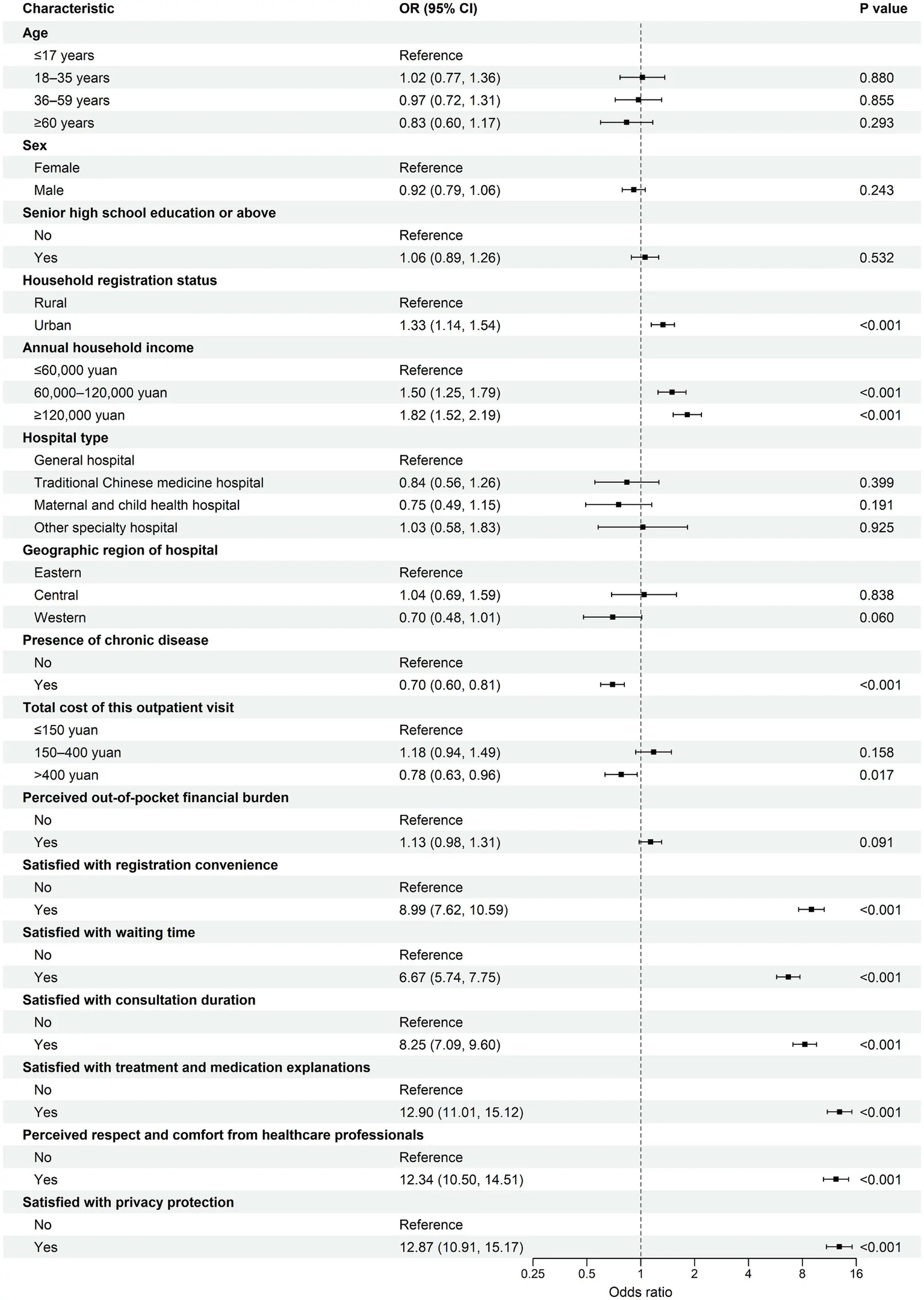
Forest plot of univariable mixed-effects logistic regression analyses for factors associated with outpatients’ endorsement of societal recognition and respect for healthcare professionals.

In crude analyses, urban household registration and higher annual household income were associated with higher endorsement, whereas the presence of chronic disease and higher outpatient visit cost were associated with lower endorsement. The strongest crude associations were observed for the six outpatient process and humanistic care indicators. Treatment and medication explanations and privacy protection ranked highest, followed by perceived respect and comfort, registration convenience, consultation duration, and waiting time (all *p* < 0.001; Figure 1). Complete estimates are presented in Figure 1.

### Multivariable analyses

As shown in Table 2, the sequential multivariable mixed-effects logistic regression models identified several factors independently associated with outpatients’ endorsement that healthcare professionals deserve societal recognition and respect. In the fully adjusted model, urban household registration remained positively associated with endorsement compared with rural registration (OR = 1.32, 95% CI: 1.08–1.62, *p* = 0.007). Higher annual household income was also associated with greater odds of endorsement, with ORs of 1.32 (95% CI: 1.05–1.67, *p* = 0.020) for 60,000–120,000 yuan and 1.52 (95% CI: 1.20–1.93, *p* < 0.001) for ≥120,000 yuan, compared with ≤60,000 yuan. In contrast, participants with chronic disease had lower odds of endorsement (OR = 0.74, 95% CI: 0.59–0.91, *p* = 0.005). Age, sex, educational attainment, hospital type, geographic region, total outpatient cost, and perceived out-of-pocket financial burden were not significantly associated with endorsement after full adjustment. In the fully adjusted model, the variance of the hospital-level random intercept was 0.305, corresponding to an ICC of 0.085 (8.5%). Thus, approximately 8.5% of the variance in the latent propensity for endorsement was attributable to differences between hospitals.

**Table 2:** Sequential multivariable mixed-effects logistic regression analyses of factors associated with outpatients’ endorsement of societal recognition and respect for healthcare professionals.

Category	Characteristic	Model 1 OR (95% CI)	Model 1 *p* value	Model 2 OR (95% CI)	Model 2 *p* value	Model 3 OR (95% CI)	Model 3 *p* value	Model 4 OR (95% CI)	Model 4 *p* value
Patient sociodemographic characteristics	Age								
	≤17 years (Ref.)	—	—	—	—	—	—	—	—
	18–35 years	1.18 (0.83, 1.67)	0.366	1.15 (0.81, 1.63)	0.439	1.38 (0.92, 2.05)	0.116	1.30 (0.84, 2.01)	0.247
	36–59 years	1.08 (0.77, 1.51)	0.650	1.04 (0.74, 1.46)	0.823	1.26 (0.85, 1.85)	0.248	1.18 (0.77, 1.81)	0.449
	≥60 years	0.97 (0.68, 1.38)	0.858	0.93 (0.65, 1.33)	0.673	1.18 (0.77, 1.78)	0.449	1.24 (0.78, 1.97)	0.362
	Sex								
	Female (Ref.)	—	—	—	—	—	—	—	—
	Male	0.92 (0.79, 1.07)	0.284	0.91 (0.78, 1.06)	0.231	1.00 (0.84, 1.19)	0.972	0.99 (0.81, 1.20)	0.888
	Senior high school education or above								
	No (Ref.)	—	—	—	—	—	—	—	—
	Yes	0.86 (0.69, 1.08)	0.193	0.86 (0.69, 1.07)	0.178	0.81 (0.63, 1.04)	0.096	0.79 (0.60, 1.04)	0.095
	Household registration status								
	Rural (Ref.)	—	—	—	—	—	—	—	—
	Urban	1.24 (1.05, 1.45)	0.009	1.23 (1.05, 1.45)	0.011	1.36 (1.13, 1.62)	<0.001	1.32 (1.08, 1.62)	0.007
	Annual household income								
	≤60,000 yuan (Ref.)	—	—	—	—	—	—	—	—
	60,000–120,000 yuan	1.45 (1.21, 1.74)	<0.001	1.45 (1.20, 1.74)	<0.001	1.47 (1.19, 1.82)	<0.001	1.32 (1.05, 1.67)	0.020
	≥120,000 yuan	1.75 (1.45, 2.11)	<0.001	1.74 (1.44, 2.10)	<0.001	1.59 (1.28, 1.96)	<0.001	1.52 (1.20, 1.93)	<0.001
Hospital characteristics	Hospital type								
	General hospital (Ref.)	—	—	—	—	—	—	—	—
	Traditional Chinese medicine hospital	—	—	0.87 (0.58, 1.28)	0.470	0.95 (0.62, 1.47)	0.828	1.05 (0.75, 1.46)	0.773
	Maternal and child health hospital	—	—	0.72 (0.48, 1.09)	0.119	0.74 (0.47, 1.18)	0.205	0.96 (0.67, 1.37)	0.811
	Other specialty hospital	—	—	0.97 (0.55, 1.69)	0.911	1.26 (0.67, 2.38)	0.476	1.58 (0.94, 2.65)	0.081
	Geographic region of hospital								
	Eastern (Ref.)	—	—	—	—	—	—	—	—
	Central	—	—	1.09 (0.72, 1.64)	0.676	1.05 (0.67, 1.66)	0.822	1.01 (0.71, 1.43)	0.965
	Western	—	—	0.78 (0.54, 1.13)	0.193	0.72 (0.48, 1.08)	0.117	0.79 (0.58, 1.08)	0.146
Health status and economic factors	Presence of chronic disease								
	No (Ref.)	—	—	—	—	—	—	—	—
	Yes	—	—	—	—	0.72 (0.60, 0.87)	<0.001	0.74 (0.59, 0.91)	0.005
	Total cost of this outpatient visit								
	≤150 yuan (Ref.)	—	—	—	—	—	—	—	—
	150–400 yuan	—	—	—	—	1.21 (0.96, 1.52)	0.111	1.15 (0.89, 1.49)	0.282
	>400 yuan	—	—	—	—	0.81 (0.66, 1.00)	0.054	1.01 (0.79, 1.27)	0.959
	Perceived out-of-pocket financial burden								
	No (Ref.)	—	—	—	—	—	—	—	—
	Yes	—	—	—	—	1.16 (0.99, 1.37)	0.073	0.90 (0.75, 1.09)	0.281
Outpatient process and humanistic care experience	Satisfied with registration convenience								
	No (Ref.)	—	—	—	—	—	—	—	—
	Yes	—	—	—	—	—	—	2.38 (1.85, 3.05)	<0.001
	Satisfied with waiting time								
	No (Ref.)	—	—	—	—	—	—	—	—
	Yes	—	—	—	—	—	—	1.59 (1.25, 2.03)	<0.001
	Satisfied with consultation duration								
	No (Ref.)	—	—	—	—	—	—	—	—
	Yes	—	—	—	—	—	—	1.86 (1.45, 2.38)	<0.001
	Satisfied with treatment and medication explanations								
	No (Ref.)	—	—	—	—	—	—	—	—
	Yes	—	—	—	—	—	—	3.08 (2.40, 3.96)	<0.001
	Perceived respect and comfort from healthcare professionals								
	No (Ref.)	—	—	—	—	—	—	—	—
	Yes	—	—	—	—	—	—	1.91 (1.47, 2.49)	<0.001
	Satisfied with privacy protection								
	No (Ref.)	—	—	—	—	—	—	—	—
	Yes	—	—	—	—	—	—	3.26 (2.55, 4.17)	<0.001

Model 1 included patient sociodemographic characteristics, including age, sex, senior high school education or above, household registration status, and annual household income. Model 2 additionally adjusted for hospital characteristics, including hospital type and geographic region of hospital. Model 3 further included health status and economic factors, including presence of chronic disease, total cost of this outpatient visit, and perceived out-of-pocket financial burden. Model 4 additionally included outpatient process and humanistic care experience, including satisfaction with registration convenience, waiting time, consultation duration, treatment and medication explanations, perceived respect and comfort from healthcare professionals, and satisfaction with privacy protection. All models included a hospital-level random intercept. OR, odds ratio; CI, confidence interval.

Outpatient process and humanistic care experience showed the strongest associations in the fully adjusted model. Satisfaction with privacy protection was associated with the highest odds of endorsement (OR = 3.26, 95% CI: 2.55–4.17, *p* < 0.001), followed by satisfaction with treatment and medication explanations (OR = 3.08, 95% CI: 2.40–3.96, *p* < 0.001), registration convenience (OR = 2.38, 95% CI: 1.85–3.05, *p* < 0.001), perceived respect and comfort from healthcare professionals (OR = 1.91, 95% CI: 1.47–2.49, *p* < 0.001), consultation duration (OR = 1.86, 95% CI: 1.45–2.38, *p* < 0.001), and waiting time (OR = 1.59, 95% CI: 1.25–2.03, *p* < 0.001). These findings suggest that, after accounting for sociodemographic, hospital, health, and economic factors, patients’ direct outpatient care experiences were most strongly related to their endorsement of societal recognition and respect for healthcare professionals. The VIFs for the six outpatient process and humanistic-care indicators ranged from 1.34 to 1.64, with all values below 5, indicating no concerning multicollinearity among these variables (Supplementary Table S3).

### Sensitivity analysis

As shown in Supplementary Figure S1, after excluding participants with neutral responses, the associations between outpatient experience indicators and endorsement remained generally consistent with the main analysis. Satisfaction with treatment and medication explanations showed the strongest association (OR = 5.03, 95% CI: 2.67–9.49, *p* < 0.001), followed by satisfaction with privacy protection (OR = 3.37, 95% CI: 1.83–6.20, *p* < 0.001), registration convenience (OR = 2.76, 95% CI: 1.51–5.02, *p* < 0.001), and consultation duration (OR = 2.23, 95% CI: 1.19–4.18, *p* = 0.012). Higher annual household income of 60,000–120,000 yuan was also associated with higher odds of endorsement (OR = 3.38, 95% CI: 1.50–7.60, *p* = 0.003).

As shown in Figure 2, conventional logistic regression models without hospital-level random intercepts yielded results largely consistent with the main mixed-effects models. Urban household registration (OR = 1.32, 95% CI: 1.08–1.61, *p* = 0.007), higher annual household income of 60,000–120,000 yuan (OR = 1.39, 95% CI: 1.10–1.75, *p* = 0.006) and ≥120,000 yuan (OR = 1.69, 95% CI: 1.34–2.13, *p* < 0.001), and absence of chronic disease (OR for chronic disease = 0.71, 95% CI: 0.58–0.88, *p* = 0.001) remained associated with endorsement. The strongest associations were again observed for privacy protection (OR = 3.38, 95% CI: 2.65–4.31), treatment and medication explanations (OR = 3.18, 95% CI: 2.48–4.09), registration convenience (OR = 2.49, 95% CI: 1.95–3.19), perceived respect and comfort from healthcare professionals (OR = 1.94, 95% CI: 1.49–2.53), consultation duration (OR = 1.88, 95% CI: 1.47–2.41), and waiting time (OR = 1.58, 95% CI: 1.24–2.02; all *p* < 0.001). As shown in Supplementary Figure S2, after excluding proxy respondents, the main findings remained generally consistent with the primary analysis. All six outpatient experience indicators remained positively associated with endorsement, with treatment and medication explanations and privacy protection showing the strongest associations. Higher annual household income of at least 120,000 yuan remained positively associated with endorsement, whereas chronic disease remained inversely associated. Overall, excluding proxy respondents did not materially alter the main conclusions.

**Figure 2:**
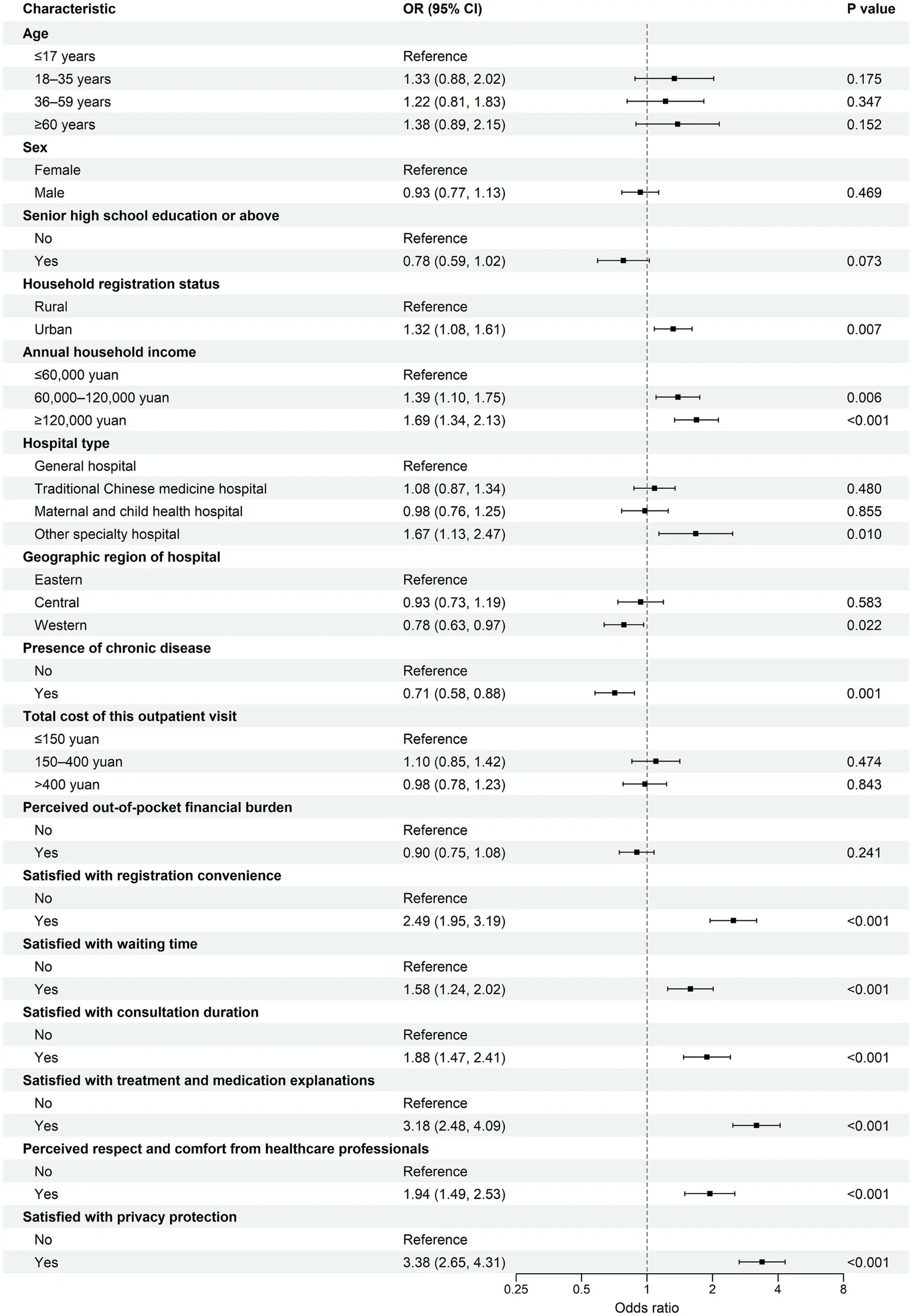
Forest plot of sensitivity analyses using conventional logistic regression models without hospital-level random intercepts for factors associated with outpatients’ endorsement of societal recognition and respect for healthcare professionals.

As shown in Figure 3, mixed-effects ordinal logistic regression models based on the original five-level response also supported the main findings. Positive outpatient experience indicators remained consistently associated with higher levels of endorsement, including satisfaction with treatment and medication explanations (OR = 2.39, 95% CI: 2.07–2.76), privacy protection (OR = 2.39, 95% CI: 2.07–2.76), perceived respect and comfort from healthcare professionals (OR = 2.04, 95% CI: 1.75–2.37), registration convenience (OR = 1.95, 95% CI: 1.70–2.25), consultation duration (OR = 1.59, 95% CI: 1.41–1.80), and waiting time (OR = 1.51, 95% CI: 1.34–1.69; all *p* < 0.001). Overall, these sensitivity analyses indicated that the direction and overall pattern of the associations were robust to alternative outcome definitions and model specifications. However, the ordinal analysis does not eliminate the ceiling effect caused by the concentration of responses at the upper end of the response scale.

**Figure 3:**
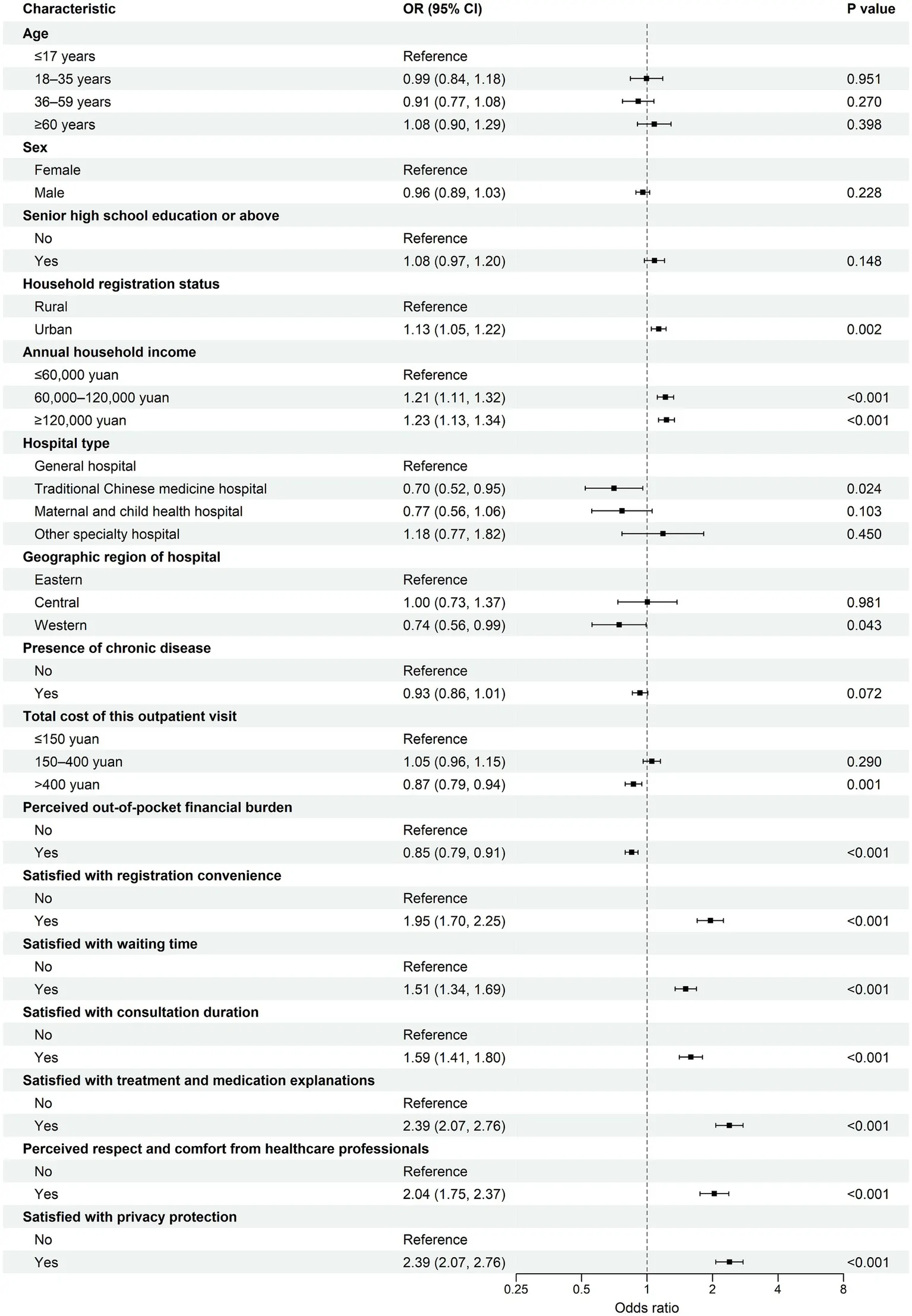
Forest plot of sensitivity analyses using mixed-effects ordinal logistic regression models with hospital-level random intercepts based on the original five-level response for factors associated with outpatients’ endorsement of societal recognition and respect for healthcare professionals.

### Latent class analysis

Latent class analysis was conducted among participants with complete data on the six outpatient experience indicators (*N* = 30,850). Latent class models with one to five classes were compared using AIC, BIC, entropy, class proportions, and interpretability. As shown in Supplementary Table S1, although AIC continued to decrease from the one-class to five-class models, BIC reached its lowest value in the four-class model (BIC = 69011.36). The four-class model showed good overall classification quality (entropy = 0.88), acceptable class sizes, and clear substantive interpretability; therefore, it was selected as the final model.

Figure 4 shows the conditional probabilities of positive responses to the six outpatient experience indicators across the four latent classes. Class 1, labeled “Overall positive experience,” was the largest group (*N* = 27,823, 90.2%) and showed consistently high positive-response probabilities across all six indicators, ranging from 0.97 to 1.00. Class 2, labeled “Humanistic-care-related dissatisfaction” (*N* = 1,115, 3.6%), showed relatively high probabilities for registration convenience, waiting time, and consultation duration, but lower probabilities for treatment and medication explanations, respect and comfort from healthcare professionals, and privacy protection. Class 3, labeled “Process-related dissatisfaction” (*N* = 1,351, 4.4%), showed relatively low probabilities for waiting time and consultation duration but high probabilities for treatment and medication explanations, respect and comfort, and privacy protection. Class 4, labeled “Overall poor experience” (*N* = 561, 1.8%), showed consistently low positive-response probabilities across all six indicators.

**Figure 4:**
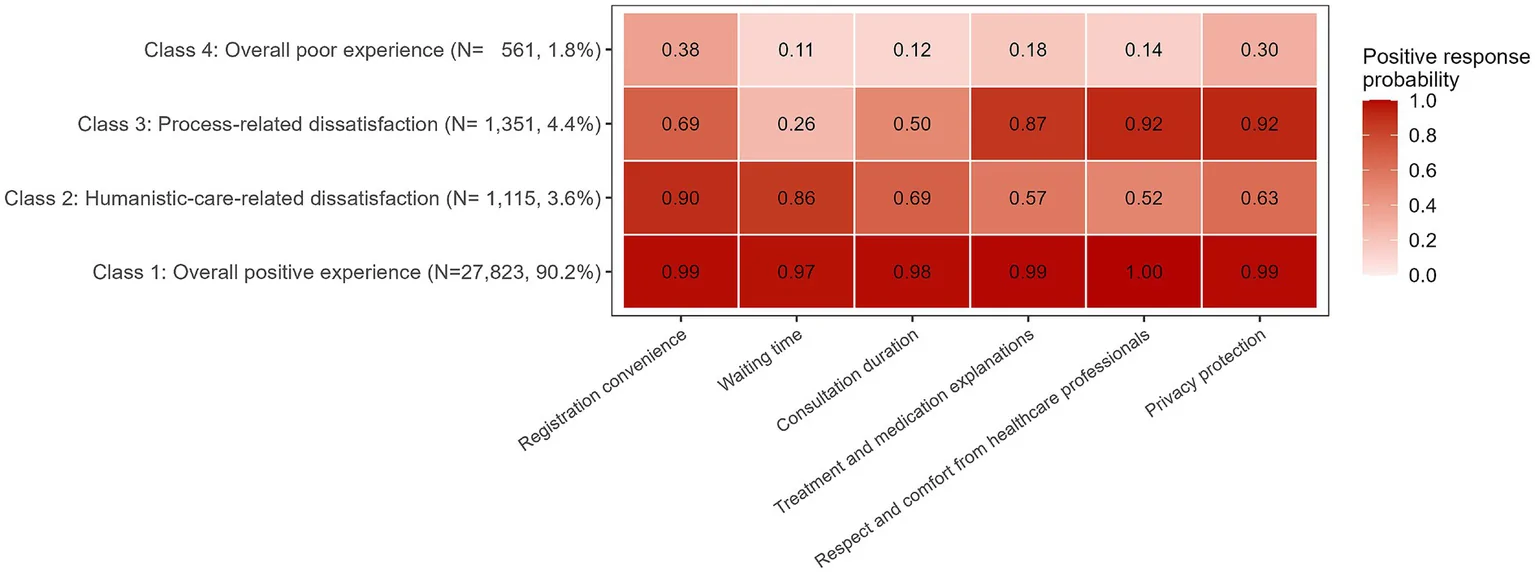
Conditional probabilities of positive responses to outpatient experience indicators across latent classes identified by latent class analysis.

As shown in Supplementary Table S2, latent outpatient experience classes were strongly associated with outpatients’ endorsement that healthcare professionals deserve societal recognition and respect. Compared with Class 1, all three dissatisfaction-related classes had markedly lower odds of endorsement after adjustment for patient sociodemographic characteristics, hospital characteristics, health status, and economic factors. The adjusted ORs were 0.097 (95% CI: 0.076–0.123, *p* < 0.001) for Class 2, 0.150 (95% CI: 0.115–0.195, *p* < 0.001) for Class 3, and 0.029 (95% CI: 0.022–0.037, *p* < 0.001) for Class 4. These results indicate that patients with poorer overall outpatient experience patterns, especially those in the “Overall poor experience” class, were substantially less likely to endorse societal recognition and respect for healthcare professionals.

## Discussion

In this national multicenter cross-sectional study of outpatients in China’s tertiary public hospitals, we found that endorsement of societal recognition and respect for healthcare professionals was high, with 97.4% of participants reporting agreement or strong agreement. On the one hand, the high endorsement rate may reflect genuine social appreciation of the social contribution and value of healthcare professionals. On the other hand, it may also partly reflect social desirability, cultural norms that favor expressing respect for the medical profession, and the normative wording of the item, which asks whether healthcare professionals “deserve” societal recognition and respect. Therefore, the 97.4% endorsement rate should be interpreted as high endorsement of this specific normative statement, rather than as a definitive measure of a broad and multidimensional construct. Despite this overall positive pattern, endorsement varied across patient characteristics and outpatient care experiences. Urban household registration and higher annual household income were associated with greater odds of endorsement, whereas chronic disease was associated with lower odds. Notably, outpatient process and humanistic care experiences showed the strongest and most consistent associations. Satisfaction with privacy protection, treatment and medication explanations, registration convenience, perceived respect and comfort from healthcare professionals, consultation duration, and waiting time were all independently associated with higher endorsement. Latent class analysis further showed that patients with dissatisfaction-related experience patterns, particularly those with overall poor outpatient experience, were substantially less likely to endorse societal recognition and respect for healthcare professionals. These findings suggest that patients’ broader social evaluation of healthcare professionals is closely tied to their concrete experiences during outpatient care. However, because of the cross-sectional design and the simultaneous assessment of outpatient experiences and endorsement, the direction of this association cannot be determined. Patients who already held more favorable attitudes toward the medical profession may also have evaluated the same outpatient experience more positively.

The high level of endorsement observed in our study is broadly consistent with the limited empirical evidence on patients’ respect-related attitudes toward healthcare professionals. A national survey in China found that only small proportions of patients reported no responsibility to respect doctors or nurses, suggesting that respect for healthcare professionals is widely recognized as part of patients’ role in healthcare relationships (22). Compared with this responsibility-oriented perspective, our study extends the evidence by focusing on outpatients’ broader endorsement that healthcare professionals, as an occupational group, deserve societal recognition and respect. Directly comparable international evidence remains scarce; however, related studies from other settings also suggest that respect in healthcare is a relational and multidimensional concept. For example, qualitative research in the United States showed that patients understood respectful care through empathy, care, autonomy, information provision, dignity, recognition of individuality, and attention to needs (23); meanwhile, research among hospital and community nurses in England distinguished between recognition respect and appraisal respect in professional healthcare relationships (8). Empirical evidence on occupational respect and prestige also suggests that healthcare professions, particularly physicians and nurses, are commonly perceived by the public as socially valuable and deserving of high professional esteem (24). These findings suggest that the profession-level normative endorsement assessed in this study may be associated with patients’ experiences of dignity, communication, and professional conduct during care. However, the outcome did not directly measure interpersonal respect, trust, reciprocity, professional prestige, or patient satisfaction, and the cross-sectional design does not establish that outpatient experiences determine patients’ endorsement.

However, the outcome distribution was highly imbalanced: 97.4% of participants endorsed the statement, whereas only 2.6% were classified as non-endorsement. This pronounced ceiling effect restricts variation in the outcome and may limit the ability to distinguish between respondents with different levels of endorsement. Because endorsement was common, the odds ratios should not be interpreted as relative risks or as direct measures of absolute differences in endorsement probability. Some relatively large odds ratios may correspond to more modest absolute differences in predicted probabilities. The ordinal sensitivity analysis supported the robustness of the overall pattern of associations, but it did not remove the interpretive limitations imposed by the ceiling effect and outcome imbalance.

Outpatient process experience may be particularly important in shaping patients’ endorsement of societal recognition and respect for healthcare professionals. In China’s tertiary hospitals, outpatient care is often delivered in a high-volume and time-constrained setting, where patients need to navigate registration, waiting, consultation, payment, examination, and medication-related procedures within a limited period. Previous national and hospital-based studies in China have shown that outpatient procedures, especially long waiting times and process management, are among the least satisfactory aspects of tertiary outpatient care (25). Studies of appointment and registration reforms have further shown that improving appointment systems and reducing waiting time can increase outpatient satisfaction, suggesting that patients’ evaluations are sensitive to the organization and efficiency of the care-seeking process (13–15). Consistent with this evidence, our study found that satisfaction with registration convenience, waiting time, and consultation duration remained independently associated with higher endorsement. These process-related experiences may matter not only because they reduce inconvenience, but also because they signal whether patients feel that their time, access to care, and need for adequate attention are being taken seriously. These process indicators should therefore be interpreted as reflecting not only individual clinician behavior but also the organizational capacity of the health system to allocate resources and deliver accessible, efficient, and appropriate outpatient services (16). Evidence on consultation duration also suggests that the length and quality of clinical encounters are closely connected with patients’ perceptions of care quality and patient-centeredness (26, 27). Therefore, optimizing outpatient processes should not be viewed solely as an efficiency-oriented management task; it may also help create a more respectful care environment in which patients are more likely to recognize the professional value of healthcare workers.

Humanistic care experience may be more directly connected to the respect-related nature of our outcome. In the fully adjusted model, satisfaction with treatment and medication explanations, perceived respect and comfort from healthcare professionals, and satisfaction with privacy protection were all strongly associated with higher endorsement, with privacy protection and treatment explanations showing the largest effect sizes. These findings are consistent with prior evidence that patient-centered care and patient experience are shaped by effective communication, respect, dignity, emotional support, information provision, involvement in care, and privacy protection (11). Studies focusing specifically on patient-perceived respect and dignity further show that patients interpret respect through concrete clinician behaviors and care environments, including listening carefully, providing understandable explanations, allowing patient input, spending enough time, protecting privacy and modesty, and attending to individual needs (28). Communication may be particularly important in this pathway, as systematic reviews have linked patient-centered communication, emotional support, and communication that values patient autonomy with stronger therapeutic relationships and higher satisfaction with care (12, 29). Privacy protection is also central to dignified care; intervention and observational studies have shown that improving ethical care environments and protecting privacy are associated with better patient perceptions and satisfaction (30, 31). From this perspective, patients’ endorsement that healthcare professionals deserve societal recognition and respect may partly reflect a reciprocal process (32): patients who feel respected, informed, comforted, and protected during care may be more likely to recognize healthcare professionals as deserving respect in return.

These associations should also be interpreted in light of the data collection context. The outcome and the main outpatient experience indicators, including treatment and medication explanations, perceived respect and comfort, privacy protection, registration convenience, waiting time, and consultation duration, were reported by the same patient in the same questionnaire and referred to the same outpatient visit. A generally favorable or unfavorable evaluation of the overall visit may therefore have influenced several responses, producing common-method and halo effects. These indicators should consequently be understood as correlated dimensions of the overall patient-reported care experience, rather than as separate objective effects on patients’ endorsement of societal recognition and respect.

Patient socioeconomic and health-related differences also deserve attention. In our fully adjusted model, urban household registration and higher annual household income were associated with greater endorsement, whereas chronic disease was associated with lower endorsement. These findings are broadly consistent with previous studies showing that sociodemographic characteristics, income, residence, perceived health status, and chronic illness are related to patients’ satisfaction, care experience, and evaluation of healthcare services (33). In the Chinese context, household registration and income may reflect differences in healthcare access, familiarity with tertiary hospital services, health literacy, and expectations toward medical care. Patients with chronic diseases often have repeated healthcare encounters, more complex care needs, and greater long-term financial and psychological burden, which may make them more sensitive to deficiencies in service delivery and interpersonal care (34). Nevertheless, the associations of sociodemographic and health-related factors were generally smaller than those of outpatient process and humanistic care indicators. This pattern suggests that patients’ endorsement of societal recognition and respect for healthcare professionals may be shaped less by background characteristics alone than by how patients experience care in concrete outpatient interactions.

The latent class analysis provides additional insight by shifting the focus from individual experience indicators to overall outpatient experience patterns (35). While the multivariable regression models showed that specific aspects of outpatient care were independently associated with endorsement, the latent class analysis demonstrated that these experiences clustered into distinct patterns among patients. The largest class was characterized by consistently positive experiences across both process-related and humanistic care indicators, whereas the other classes reflected humanistic-care-related dissatisfaction, process-related dissatisfaction, or overall poor experience. Patients in all dissatisfaction-related classes had markedly lower odds of endorsing societal recognition and respect for healthcare professionals, with the lowest odds observed among those with overall poor outpatient experience. This suggests that patients’ social evaluation of healthcare professionals may be shaped not only by isolated service encounters, but also by the accumulation and combination of multiple care experiences. Importantly, both process-related and humanistic-care-related dissatisfaction were associated with lower endorsement, indicating that efficient outpatient management and respectful interpersonal care may jointly contribute to patients’ recognition of healthcare professionals’ social value.

These findings have implications not only for individual clinicians but also for hospital management and health-system leaders. Relevant areas for organizational and system-level attention include appointment and registration organization, waiting-time management, privacy protection, adequate consultation time, and support for clear treatment and medication communication. Such efforts may include improving workflow design, staffing and scheduling arrangements, privacy infrastructure, and communication support within outpatient services. These policy implications should be understood as areas for organizational attention rather than as evidence that specific interventions would causally increase patients’ recognition and respect for healthcare professionals.

This study has several strengths. First, it used data from a large national multicenter survey covering tertiary public hospitals across all 31 provincial-level administrative regions of mainland China, providing a broad empirical picture of outpatients’ endorsement of societal recognition and respect for healthcare professionals. Second, the analysis incorporated both patient-level and hospital-level information and accounted for the clustering of patients within hospitals through mixed-effects models, which better reflected the hierarchical structure of the data. Third, the findings were supported by several sensitivity analyses, including alternative outcome definitions and model specifications. Finally, beyond conventional variable-centered regression, the latent class analysis provided a complementary person-centered perspective, showing how different combinations of outpatient process and humanistic care experiences were related to patients’ broader recognition of healthcare professionals.

This study has several limitations. First, its cross-sectional design precludes causal inference, and the direction of the association between outpatient experience and endorsement cannot be determined. Second, the study was conducted in tertiary public hospitals, which may limit the generalizability of the findings to primary care institutions, private hospitals, inpatient settings, or other healthcare systems. Although the participating hospitals covered all 31 provincial-level administrative regions of mainland China, this geographic coverage does not imply that the patient sample was a nationally representative probability sample of all outpatients in China. Non-response and recruitment within hospitals may have introduced selection bias. Data were collected in the first quarter of 2021 during the COVID-19 pandemic, when public perceptions of healthcare professionals and healthcare-seeking behavior may have differed from those in other periods. Third, the outcome was measured using a single item capturing a profession-level normative attitude rather than a multidimensional measure of societal appreciation, interpersonal respect, professional prestige, trust, reciprocity, or patient satisfaction. The high endorsement rate (97.4%) also indicates a pronounced ceiling effect and may make the odds ratios sensitive to the small non-endorsement group; social desirability may have further contributed to this pattern. Fourth, latent class membership was assigned according to the highest posterior probability. Although the four-class model had good classification quality (entropy = 0.88), modal assignment does not eliminate individual-level classification uncertainty and may have introduced some misclassification. Finally, the outcome and predictors were reported by the same patient or proxy respondent in the same questionnaire and referred to the same outpatient visit, creating potential recall, social desirability, common-method, and halo biases. Therefore, the individual predictors should not be interpreted as separate objective effects. Future studies should use longitudinal designs, multidimensional validated instruments, objective service indicators, and samples from primary care, private hospitals, and other healthcare systems to better distinguish pre-existing attitudes from evaluations formed during care.

## Conclusion

In this national multicenter study of outpatients in China’s tertiary public hospitals, most participants endorsed that healthcare professionals deserve societal recognition and respect. Endorsement was associated with sociodemographic and health-related characteristics and was more strongly associated with concrete outpatient experiences, particularly privacy protection, treatment and medication explanations, registration convenience, perceived respect and comfort, consultation duration, and waiting time. Patients with poorer overall experience patterns had lower odds of endorsement. These findings suggest that patients’ evaluations of the social value of healthcare professionals are related to both the organizational and human dimensions of outpatient care, although the direction of these associations cannot be determined. Further research using multidimensional measures and longitudinal designs is needed.

## Data Availability

The datasets analyzed in this study are not publicly available due to data sharing restrictions and the need to protect participant confidentiality. The data were derived from the fifth National Third-Party Evaluation of the Action Plan for Further Improving Healthcare Services. Access to the data is subject to approval by the relevant research team and data management authorities. Requests to access the datasets may be directed to the corresponding author and will be considered on reasonable request. Requests to access these datasets should be directed to Yuanli Liu, liuyuanli_pumc@163.com.
