# Supplementary material for: Outpatients’ endorsement of societal recognition and respect for healthcare professionals in China’s tertiary public hospitals: a national multicenter cross-sectional study
Source: Front Public Health. 2026 Oct 2;14:1893761. doi: 10.3389/fpubh.2026.1893761 (PMC13672632; doi:10.3389/fpubh.2026.1893761)
Supplement: Supplementary file 1 [file Data_Sheet_1.docx]

# Supplementary materials

Table S1. Fit indices for latent class models of outpatient experience patterns

| **Number of classes** | **AIC** | **BIC** | **Entropy** | **Minimum latent class proportion, %** | **Maximum latent class proportion, %** |
| --- | --- | --- | --- | --- | --- |
| 1 | 84406.56 | 84456.58 | NA | 100.00 | 100.00 |
| 2 | 70163.85 | 70272.23 | 0.90 | 9.99 | 90.01 |
| 3 | 69307.22 | 69473.95 | 0.86 | 4.89 | 87.22 |
| 4 | 68786.27 | 69011.36 | 0.88 | 1.92 | 86.96 |
| 5 | 68744.54 | 69028.00 | 0.89 | 1.53 | 87.51 |

Note: The four-class model was selected based on the lowest BIC, good entropy, acceptable latent class proportions, and interpretability. Entropy was not calculated for the one-class model because classification uncertainty is not defined when only one latent class is specified. AIC, Akaike information criterion; BIC, Bayesian information criterion.

Table S2. Association between latent outpatient experience classes and outpatients’ endorsement of societal recognition and respect for healthcare professionals

| **Latent class** | **N (%)** | **Adjusted OR (95% CI)** | **P value** |
| --- | --- | --- | --- |
| Class 1: Overall positive experience | 27,823 (90.2%) | Reference | — |
| Class 2: Humanistic-care-related dissatisfaction | 1,115 (3.6%) | 0.097 (0.076, 0.123) | <0.001 |
| Class 3: Process-related dissatisfaction | 1,351 (4.4%) | 0.150 (0.115, 0.195) | <0.001 |
| Class 4: Overall poor experience | 561 (1.8%) | 0.029 (0.022, 0.037) | <0.001 |

Note: Estimates were derived from mixed-effects logistic regression models with a hospital-level random intercept. The model was adjusted for age, sex, senior high school education or above, household registration status, annual household income, hospital type, geographic region of hospital, presence of chronic disease, total cost of this outpatient visit, and perceived out-of-pocket financial burden. OR, odds ratio; CI, confidence interval.

Table S3. Multicollinearity diagnostics for the six outpatient process and humanistic-care indicators

| **Indicator** | **VIF** | **VIF 95% CI** | **Tolerance** |
| --- | --- | --- | --- |
| Satisfaction with registration convenience | 1.34 | 1.31-1.36 | 0.75 |
| Satisfaction with waiting time | 1.52 | 1.50-1.55 | 0.66 |
| Satisfaction with consultation duration | 1.59 | 1.56-1.62 | 0.63 |
| Satisfaction with treatment and medication explanations | 1.56 | 1.53-1.59 | 0.64 |
| Perceived respect and comfort from healthcare professionals | 1.64 | 1.62-1.67 | 0.61 |
| Satisfaction with privacy protection | 1.41 | 1.39-1.44 | 0.71 |

Note: VIF, variance inflation factor; CI, confidence interval. VIFs were derived from the fully adjusted mixed-effects logistic regression model. All VIFs were below 5, indicating no concerning multicollinearity.

Figure S1. Forest plot of sensitivity analyses excluding neutral responses using mixed-effects logistic regression models for factors associated with outpatients’ endorsement of societal recognition and respect for healthcare professionals

Figure S2. Forest plot of the sensitivity analysis excluding proxy respondents using a mixed-effects logistic regression model for factors associated with outpatients' endorsement of societal recognition and respect for healthcare professionals
